# School Functioning Among Norwegian Children With a History of Maltreatment

**DOI:** 10.1177/08862605251351669

**Published:** 2025-07-11

**Authors:** Sofie Øvstebø Næss, Børge Sivertsen, Mari Hysing, Anders Dovran, Stian Tobiassen, Gertrud Sofie Hafstad, Kaia Kjørstad

**Affiliations:** 1 Regional Centre for Child and Youth Mental Health and Child Welfare, NORCE Norwegian Research Centre, Bergen, Norway; 2 Department of Psychosocial Science, Faculty of Psychology, University of Bergen, Norway; 3 Department of Health Promotion, Norwegian Institute of Public Health, Bergen, Norway; 4 Department of Research & Innovation, Helse Fonna HF, Haugesund, Norway; 5 Stine Sofie’s Foundation & the Stine Sofie Center, Grimstad, Norway; 6 Norwegian Center for Violence and Traumatic Stress Studies, Oslo, Norway

**Keywords:** child maltreatment, multivictimization, school functioning, school performance, special education, school absence

## Abstract

Childhood maltreatment increases the risk of adverse outcomes in various areas, such as somatic and mental health. This may also lead to poorer academic achievement, but few studies have been conducted among children in primary school. The study aimed to investigate school functioning in a sample of Norwegian children subjected to maltreatment and the association with multivictimization (i.e., exposure to three or more types of maltreatment). Parent-reported data on behalf of 192 children (ages 6–12 years) were collected from the Stine Sofie Center, a support and coping center for children exposed to maltreatment. School functioning was assessed through parent-reported school performance, special education status, and school absences. In total, 15.6% of the children were reported to perform very poorly in at least one subject. Approximately one-fourth (27.1%) received special education, and 10.0% had been absent from school for at least 8 days in the last month. Multivictimization was associated with an increased likelihood of poorer school performance (*OR* 3.15, 95% CI [1.42, 7.03]) and higher levels of school absences (*OR* 2.75, 95% CI [1.26, 8.68]) but not enrollment in special education. Children who have experienced maltreatment, especially multivictimization, face a high risk of poor school performance and high absenteeism. These results underscore the urgent need to better understand the effects of maltreatment and multivictimization on school functioning to develop more effective intervention and support strategies. Future research should continue to examine the long-term educational impacts of maltreatment and evaluate the effectiveness of intervention programs. Ensuring that maltreated children receive the necessary educational support is crucial in helping them succeed academically and overcome the challenges posed by early adversity.

## Introduction

Child maltreatment refers to abuse experienced by children under 18 years, encompassing physical and/or emotional ill-treatment, sexual abuse, and neglect that causes potential or actual harm to the child (World Health Organization, 2022). Unfortunately, experiencing maltreatment is not uncommon. Globally, an estimated 18% of children experience neglect, 36% psychological abuse, 23% physical abuse, and 13% sexual abuse (Stoltenborgh et al., 2015). In Norway, the UEVO study (*N* = 9,240; ages 12–16) reported 14% neglect, 20% psychological abuse, 19% physical abuse, and 13% to 17% witnessing violence against a parent (Hafstad & Augusti, 2019).

Exposure to maltreatment has been consistently linked to long-term cognitive, emotional, and behavioral difficulties (Gilbert et al., 2009). The Developmental Psychopathology Framework (Cicchetti & Rogosch, 2002) offers a comprehensive perspective on how childhood maltreatment disrupts learning and school attendance. This framework emphasizes how early adversity interacts with biological, psychological, and environmental factors across developmental stages, shaping children’s ability to regulate emotions, sustain attention, and engage socially. Maltreatment experiences can interfere with executive functioning, including working memory and cognitive flexibility (Clinchard et al., 2024) and attention regulation and stress response systems (McLaughlin et al., 2014), which together make it harder for children to engage in learning and adapt to structured school environments.

Studies consistently show that maltreated children perform worse academically than their non-maltreated peers. A meta-analysis of 32 studies found that children aged 3 to 18 years with a history of maltreatment scored significantly lower on academic assessments (McGuire & Jackson, 2018), a finding reinforced by a larger meta-analysis of 59 studies (Qu et al., 2024). Neglect and psychological abuse appear to be particularly strong predictors of academic difficulties (Li et al., 2022), while exposure to violence has been linked to lower grade point averages (Hurt et al., 2001). Research on adverse childhood experiences (ACEs) has also demonstrated that early adversity increases the likelihood of academic disengagement, contributing to lower school achievement (Blodgett & Lanigan, 2018; Stewart-Tufescu et al., 2022; Tan et al., 2017). Moreover, childhood adversity has been associated with reduced educational attainment, including decreased likelihood of high school graduation (Morrow & Villodas, 2018; Stempel et al., 2017).

School absenteeism strongly predicts school disengagement and dropout (De Ridder et al., 2012; Freudenberg & Ruglis, 2007). For children with a history of maltreatment, school attendance can provide structure, stability, and access to supportive adults (Chafouleas et al., 2019). However, absenteeism may also signal academic and mental health struggles, including trauma-related school avoidance, anxiety, and social disengagement (Kearney et al., 2023). A study from Western Australia found that reduced school attendance predicted lower academic achievement over time (Maclean et al., 2016). Additionally, findings from the UEVO study suggest that maltreated adolescents, particularly those experiencing multivictimization, have higher rates of absenteeism (Hafstad & Augusti, 2019). Research on ACEs and school attendance further highlights that exposure to multiple adversities—including parental substance abuse, community violence, and financial instability—is associated with an increased likelihood of chronic absenteeism (Stempel et al., 2017).

Given the increased academic challenges maltreated children face, many require special education support. A large U.S. study of 8- to 9-year-olds (*N* = 732,838) found that 21.2% of children with a history of maltreatment received special education, compared to 11.6% of non-maltreated children (Ryan et al., 2018). Similar findings were reported in a population-based study of 7- to 16-year-olds, where 24.2% of maltreated children required special education services compared to 11.4% of the general population (Jonson-Reid et al., 2004). While special education services provide crucial academic support, they also reflect increased learning difficulties rather than being a direct consequence of maltreatment severity. Early identification and trauma-informed educational approaches have been found to improve educational outcomes for maltreated children (Bethell et al., 2014; Stewart-Tufescu et al., 2022). However, further research is needed to determine how special education interacts with multivictimization and whether certain subgroups of maltreated children are at greater risk of requiring educational accommodations.

Multivictimization—the experience of multiple types of victimization across different contexts—is a well-documented phenomenon in child maltreatment research. Children who experience one form of violence are at increased risk of experiencing others, creating a cumulative burden of adversity (Holt et al., 2007). A dose–response relationship has been observed between the number of ACEs and poorer educational outcomes, reinforcing the importance of examining how exposure to multiple types of maltreatment impacts school functioning (Blodgett & Lanigan, 2018). Research suggests that a safe and supportive school environment may help buffer some of these effects, potentially mitigating the negative consequences of trauma exposure (Bethell et al., 2014). However, the specific relationship between multivictimization and school functioning remains underexplored.

Despite evidence linking maltreatment to poor academic outcomes, absenteeism, and special education needs, few studies have examined these relationships in younger children. This study aims to address this gap by investigating school performance, absenteeism, and special education needs among Norwegian children aged 6 to 12 years, with a specific focus on the impact of multivictimization.

## Methods

The Triple-S study is an ongoing longitudinal study with a multi-informant design, which collects data from children and adolescents who have experienced maltreatment, their parents, and administrative sources (Schonning et al., 2021). The overall aim of the study is to examine the extent and consequences of childhood maltreatment.

### Participants and Procedure

Participants are recruited from the Stine Sofie Center, a support and coping center for children and adolescents exposed to physical, psychological, or sexual abuse, as substantiated by health personnel, child protective services, crisis centers, or other official instances. The center aims to provide care-based support in a secure environment during recovery after experiencing maltreatment (Stine Sofie Senteret, 2018).

The present study is based on parent-reported data for children aged 6 to 12 years who are enrolled in school. In Norway, most children attend public schools, and all children start school in August, the year they turn six. To be included, the parents needed to be able to complete the questionnaire in Norwegian. According to Norwegian law, foster parents are not eligible to consent to participation in research on behalf of minors and are not included in the current study (Helseforskningsloven, 2008).

Between January 2021 and December 2023, 324 children under 12 years of age attended the center with their parent(s), and 174 were included in the study, yielding a response rate of 53.7%. Of these, 38 children were excluded from this study as they had not yet started school. Additionally, parents of children who stayed at the center between 2016 and 2020 were retrospectively invited to participate. Of these, 56 were accepted, yielding a total sample size of 192 participants in the present study. Unfortunately, due to uncertainties related to how many changed their contact information or moved since their stay, we could not estimate a response rate for this phase.

### Ethics

The study was approved by The Regional Committees for Ethics in Medical and Health Research in the southeastern region of Norway (#95445). Informed consent was obtained through a secure web-based platform. All data were treated in accordance with the EU General Data Protection Regulation (GDPR). Parents were informed that participation was voluntary and that they could withdraw at any time without any reason. Given the potentially burdensome nature of the study’s topic, health personnel were made available to the participants and offered support as needed, either onsite at the center or via telephone.

### Measures

#### Sociodemographic Characteristics

Children’s age and sex at birth were extracted from their Norwegian 11-digit personal identity number. The parents reported country of origin, coded as “Child or parent born abroad” if either the child or both parents were born abroad. The current living situation was assessed by checking off several options (e.g., with biological/adoptive mother/father, siblings, a new partner, or in an institution), which were later merged to ensure anonymity. Living with mother/father includes biological/adoptive mother/father, as adoption leads to the exact status of caretaking as biological parenthood. Living with siblings included biological/half/adoptive siblings. The parents’ education was assessed as the highest level completed, categorized as primary education, secondary education, or college/university.

#### School Functioning

School functioning was evaluated by using school performance, special education enrollment, and recent school absences as indicators. School performance was assessed based on parents’ evaluation of the child’s performance in English, mathematics, and Norwegian, scored as (1) *very poor*, (2) *poor*, (3) *good*, or (4) *very good*. For this study, very poor performance in at least one subject was defined as *poor* performance, while all other options were considered *good*. School absence was assessed by indicating how many days and single hours the child was absent from school in the last month. The response options ranged from 0 to 25 days and 0 to 100 single hours. Hours and days were merged and categorized as school absences in days. A dichotomous variable was created using the 90th percentile as a cutoff and used in the analyses. A high level of school absence was defined as being absent from school 8 or more days in the last month. Lastly, special education status was assessed by asking the parents, “Has a decision been made regarding the child’s special education?” In Norway, such a decision is made following an expert assessment by the Educational and Psychological Counselling Service, which determines whether the child requires extra assistance to achieve satisfactory learning outcomes.

#### Child Maltreatment

Five categories of childhood maltreatment were assessed: (a) neglect, (b) psychological abuse, (c) physical abuse, (d) sexual abuse, and (e) witnessing violence.

Neglect was assessed by six items derived from a validated instrument used in the ACE study, adapted for use in Norwegian according to the UEVO study (Hafstad et al., 2020). The items were assessed on a 5-point scale ranging from (1) *never*, (2) *rarely*, (3) *sometimes*, (4) *often* to (5) *very often/always*, and covered whether the child experienced (1) *having enough to eat*, (2) *having to wear dirty clothes*, (3) *their parents being too drunk or high to take care of the child*, (4) *being taken to a doctor when needed*, (5) *that someone in the family made the child feel important or special*, and (6) *that the child felt that someone cared for it*. For the present study, the child was categorized as having experienced neglect if the parent indicated *never* or *rarely* on items 1, 4, 5, and 6 or *often* or *very often/always* on items 2 and 3.

Psychological abuse was assessed by 7 items from the Parent-Child Conflict Tactics Scale (Straus et al., 1998) adapted for use in Norwegian according to the UEVO study(Hafstad et al., 2020). The items were assessed on a 4-point scale ranging from (1) *never*, (2) *once*, (3) *sometimes*, (4) *often*, and covered whether the child has experienced (1) *being made fun of in a hurtful way*, (2) *being told they are stupid or unable to do anything*, (3) *to be threatened with abandonment or being sent away*, (4) *to be threatened with being beaten or hurt*, (5) *to be locked out of home*, (6) *to be locked inside a basement*, and (7) *that anyone threatened to harm the family’s pet*. For the purpose of the child, it was categorized as exposed to psychological abuse if the parent indicated *sometimes* or *often* on any item.

Physical abuse was assessed by a *yes* or *no* response to the questions (a) “Has the child been subjected to physical violence or beaten up by an adult?” and (b) “Has the child been subjected to physical violence or beaten up by a peer or youth (under 18 years of age)?” If the parent indicated *yes* to either of these questions, the child was categorized as exposed to physical abuse.

Sexual abuse was assessed by a *yes* or *no* response to the questions (a) “Has the child experienced rape or attempted rape?” and (b) “Has the child been the victim of other sexual acts like unwanted and inappropriate touching and feeling?” As for physical abuse, both questions were asked twice to differentiate between acts carried out by adults and peers/adolescents. If the parent indicated *yes* or *no* to either of these questions, the child was categorized as exposed to sexual abuse.

Witnessing violence was assessed using 6 items previously used in the UEVO study (Hafstad et al., 2020). The items were assessed on a 4-point scale, ranging from (1) *never*, (2) *once*, (3) *a few times*, (4) *many times* and addressed if the child had witnessed inmate partner violence, including the acts of being (1) *yelled at*, (2) *ridiculed*, (3) *pushed or shaken violently*, (4) *hit*, (5) *severely beaten*, or (6) *exposed to other forms of violence*. The items were asked twice to differentiate between violence against the mother and the father. For the present study, the child was categorized as exposed if the parent indicated *a few times* or higher on any item.

#### Multivictimization

A multivictimization variable was created by summarizing the number of different types of maltreatment each child had experienced and dichotomized into two categories: (a) being exposed to one or two different types of maltreatment or (b) being exposed to three or more different types of maltreatment.

### Statistical Procedures

IBM SPSS Statistics (version 29; IBM Corp., Armonk, NY, USA) for Mac was utilized for all statistical analyses. Descriptive analyses were conducted to characterize the sample and provide statistics on school functioning. These analyses calculated means and frequencies for continuous and categorical variables, respectively, along with corresponding standard deviations and 95% confidence intervals. Pearson’s chi-square tests were employed to explore the association between multivictimization and school functioning. Logistic regression analyses were used to produce odds ratios. To assess potential confounding, we tested whether socioeconomic factors, including household income, as well as age and sex, were associated with both multivictimization and school performance. As no significant associations were found with both variables, these factors were not included as covariates in the regression models. To analyze school absenteeism as a continuous variable, independent samples *t*-tests were conducted to compare the number of absent days and total hours missed between children exposed to multivictimization and those exposed to fewer types of maltreatment. We utilized the UpSetR-package in R (R Foundation for Statistical Computing, Vienna, Austria) via RStudio (Posit, PBC, Boston, MA, USA) to visually illustrate the prevalence and combinations of maltreatment types. This visualization helped elucidate complex patterns of maltreatment comprehensively and interpretably, highlighting the frequent combinations of maltreatment types encountered within the sample. Additional figures illustrating the results were generated in Microsoft Excel (version 16.82; Microsoft Corp., Redmond, WA, USA). Given that missing data were minimal (e.g., only 1.6% missing for school performance and 0.4% for absenteeism), listwise deletion was used to handle missing values.

## Results

### Characteristics of the Sample

Table 1 outlines the key sociodemographic characteristics of the sample. The children had a mean age of 9.7 years (*SD* = 1.5). A total of 107 children (55.7%) were girls, and 85 (44.3%) were boys. Most children were born in Norway and had parents who were also born in Norway, while 14.6% were either born abroad or had at least one parent born abroad.

**Table 1.** Sociodemographic Characteristics of the Study Sample (*N* = 192).

Baseline characteristics	%	*n*
Age, years		
6	3.1	6
7	15.1	29
8	11.5	22
9	25.0	48
10	19.3	37
11	22.9	44
12	3.1	6
Sex at birth		
Female	55.7	107
Male	44.3	85
Child or parent born abroad		
Born in Norway	85.4	164
Born abroad	14.6	28
Current living situation^a^		
Living with mother	91.7	176
Living with father	17.7	34
Living with both parents	11.5	22
Living with stepparent	14.6	28
Living with siblings or half-siblings	28.1	54
Parental education		
Primary	8.9	13
Secondary	38.5	57
College/University	52.7	78
Household income, Norwegian krone^b^		
Under 250,000	10.5	15
250,000–499,000	42.0	60
500,000–749,000	25.2	36
750,000 or more	22.3	32
Parental economic well-being^c^		
Very poor	8.9	14
Poor	21.5	34
Neither	41.8	66
Good	24.1	38
Very good	3.8	6

a Living conditions do not add to 100% as multiple answers were possible.

b Missing data 25.5% (*n* = 49).

c Missing data 17.7% (*n* = 34).

A significant majority (91.7%) lived with their mother. Among them, 17.7% lived with both parents, and 14.6% lived with a stepparent. Regarding parental education, half of the parents had completed college or university education, while the remainder had primary (8.9%) or secondary (35.8%) education.

### Maltreatment Experiences Within the Study Sample

**Figure 1.**
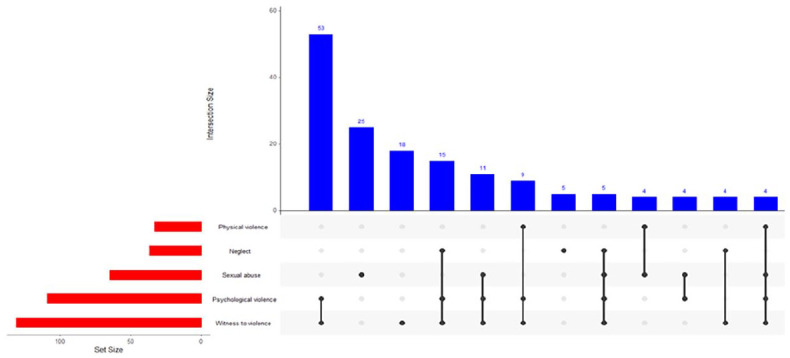
The most prevalent types and combinations of maltreatment. *Note.* Single black dots represent individual types of maltreatment, while connecting dots represent combinations of multiple types of maltreatment. Set size refers to the total number of cases of a particular maltreatment type (irrespective of combinations), while intersection size indicates the number of cases that share unique maltreatment types (incl. combinations).

As illustrated in Figure 1, witnessing violence and psychological/physical abuse were the most prevalent types of maltreatment. The most frequently reported combination was psychological abuse and witnessing violence (27.6%), followed by sexual abuse alone (13.0%). Two-thirds of the children (66.7%) had experienced more than one type of maltreatment, with 28.1% exposed to three or more different types.

### School Performance

**Figure 2.**
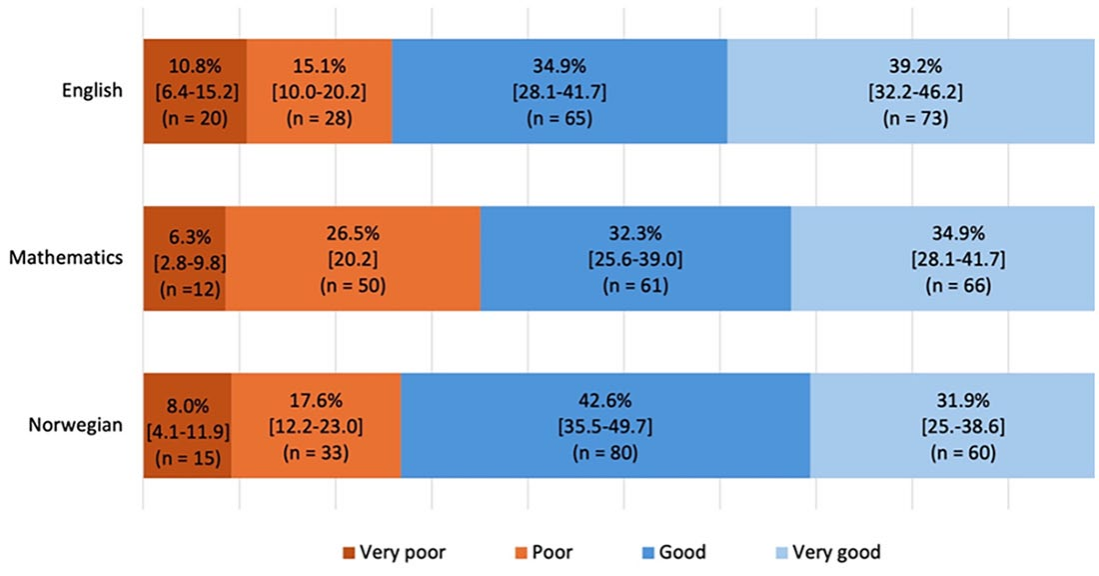
Parent-reported school performance of the children.

Figure 2 presents parent-reported school performance in English, mathematics, and Norwegian. While most children were reported to have poor, good, or very good performance across all three subjects, a notable 15.6% (*n* = 30) were reported to perform very poorly in at least one subject. The proportion of children with poor performance was the highest in mathematics. Additionally, older children were more likely to have very poor performance (χ² = 10.45, *p* = .005).

### School Absences

On average, children were absent for 3 days (*M* = 3.2; *SD* = 4.9) in the past month. The majority (60.7%) had 2 or fewer days of absence, while 29.3% were absent for 2 to 7 days, and 10.0% had 8 or more days of absence.

### Special Education Status

Approximately one-fourth of the children (27.1%) had a formal decision for special education, and among these, 92.2% received support across multiple subjects. A significant sex difference was observed, with boys more likely than girls to receive special education (37.6% vs. 18.7%, χ² = 8.62, *p* = .003).

### School Functioning and Multivictimization

The results in Table 2 show significant differences in school performance and absenteeism based on multivictimization status. Children exposed to three or more types of maltreatment were 3.15 times more likely (95% CI [1.42, 7.03]) to perform very poorly in at least one subject compared to those with fewer maltreatment exposures. Additionally, children experiencing multivictimization had 3.31-fold increased odds (95% CI [1.26, 8.68]) of having high levels of school absences (more than 8 days per month).

**Table 2.** Differences in School Functioning Between Those Exposed to Two or Less Types of Maltreatment (*n* = 123) and Those Exposed to Three or More Types (*n* = 69).

Types of Abuse	One or Two, % (*n*)	Three or More, % (*n*)	*p*-Value	Odds Ratio [95% CI]
School performance			.007	3.15 [1.42, 7.03]
Good	89.1 (123)	72.2 (39)		
Poor	10.9 (15)	27.8 (15)		
School absence			.016	2.75 [1.26, 8.68]
Low	93.4 (128)	81.1 (43)		
High	6.6 (9)	18.9 (10)		
Special education			.070	1.95 [0.99, 3.85]
No	76.8 (106)	63.0 (34)		
Yes	23.2 (32)	37.0 (20)		

When treating school absences as a continuous variable, the same pattern emerged. Children exposed to multivictimization had an average of 3.6 days of absence (*SD* = 5.2), compared to 2.2 days (*SD* = 2.7, *p* = .014) in the lower exposure group. Similarly, children in the multivictimization group missed 8.3 hr (*SD* = 18.5), compared to 2.3 hr (*SD* = 9.4, *p* = .003).

### School Performance and Special Education

Contingency table analyses examined the association between poor school performance and special education status. Children who performed very poorly in at least one subject were 19.1 times more likely (95% CI [7.16, 51.15]) to have a decision for special education compared to their peers without reported poor performance.

## Discussion

The main aim of the present study was to investigate school functioning in a population of children with a history of maltreatment, with particular emphasis on multivictimization (i.e., exposure to three or more types of abuse). Consistent with prior research on multivictimization (Finkelhor et al., 2014; Lev-Wiesel et al., 2019), our findings confirm that maltreatment rarely occurs in isolation. Two-thirds of the children in our sample had experienced multiple types of maltreatment, with witnessing violence and psychological or physical abuse being the most common, followed by sexual abuse and neglect. Educational outcomes in this sample were notably poor. One in 7 children performed very poorly in at least 1 subject, 1 in 4 received special education, and 1 in 10 had high absenteeism rates. Multivictimization was associated with more than a threefold increase in the odds of very poor school performance and nearly a threefold increase in school absences. While no significant association was found between multivictimization and special education, the result approached significance (*p* = .070), suggesting that the severity of maltreatment may contribute to educational support needs. These findings underscore the need for a deeper understanding of the academic challenges faced by maltreated children and the development of targeted interventions to support them in school.

### Educational Challenges Among Maltreated Children

A significant proportion of children in our study struggled academically. Fifteen percent performed very poorly in at least one subject, which aligns with previous studies demonstrating that maltreated children are at a significantly higher risk of academic underachievement (Li et al., 2022; McGuire & Jackson, 2018). While direct comparisons are difficult due to differences in grading systems, our results are consistent with prior research showing that maltreated children often experience difficulties in key academic areas (McGuire & Jackson, 2018). For example, our findings show that the proportion of children with poor performance was the highest in mathematics, possibly reflecting the impact of maltreatment on memory skills and executive functioning skills, which are particularly important for success in this subject. These difficulties are important to address, as they may persist across the lifespan and impact other areas of daytime functioning into adulthood (Letkiewicz et al., 2021).

Multivictimization was strongly linked to poor school performance, supporting findings from broader research on its negative impact on cognitive and emotional functioning. Studies have shown that children exposed to multiple types of maltreatment are more likely to develop emotional dysregulation (Kim & Cicchetti, 2010), which in turn is associated with higher levels of externalizing and internalizing symptoms. These symptoms have been linked to lower academic achievement and school adaptation (Pedersen et al., 2019).

Approximately one in four children (27.1%) in our study received special education, a rate significantly higher than that of the general Norwegian primary school population, where special education rates range from 3.8% in first grade to 10.5% in seventh grade (Utdanningsdirektoratet, 2023). This aligns with international research showing that maltreated children are about twice as likely to receive special education as their non-maltreated peers (Jonson-Reid et al., 2004; Ryan et al., 2018). While we did not find a significant association between multivictimization and special education, the result was approaching significance (*p* = .070), suggesting that the severity of maltreatment may contribute to the likelihood of receiving special education, even though the statistical power of this study was limited by the sample size. Additionally, the included sample consisted of relatively young children and some difficulties that impact school functioning (e.g., Attention Deficit Hyperactivity Disorder and learning difficulties) typically identified later in childhood (Elklit et al., 2016; Stern et al., 2018). Therefore, the observed trend may indicate that the association between multivictimization and special education needs becomes more evident with age, and that a larger proportion of these children may receive special education in the future.

School absenteeism was notably high among some children in our study. One in 10 had been absent for more than 8 days in the past month, while nearly one in three missed between 2 and 8 days. Children exposed to multivictimization not only had a greater likelihood of high absenteeism but also showed consistently higher average absences, both in full days and total hours missed. These findings suggest that children experiencing multiple forms of maltreatment are at an increased risk of disengagement from school, even at lower levels of absenteeism, which may accumulate over time and contribute to long-term academic difficulties. While comprehensive national data on primary school attendance in Norway are lacking, national registries for lower secondary school report a median of 8 absent days per school year in 2023 to 2024 (Norwegian Directorate for Education and Training, 2024), suggesting that absenteeism in our sample was particularly high. Our results align with studies on older adolescents (ages 12–16 years), which show that maltreated children have higher rates of school absences compared to non-maltreated peers (Hafstad & Augusti, 2019; Slade & Wissow, 2007). However, the relationship between maltreatment and school attendance may be moderated by the child’s age. School absence among the children in our sample is likely to be more influenced by parental factors compared with school absences among adolescents, as young children may require more parental assistance to get themselves ready for school in the morning. If, for example, a parent struggles with their mental or physical health, this could also impact their ability to facilitate school attendance. Regardless of the underlying mechanisms, absenteeism is a well-documented predictor of long-term academic disengagement (De Ridder et al., 2012; Freudenberg & Ruglis, 2007), emphasizing the importance of early identification and interventions for at-risk children.

### Theoretical and Mechanistic Perspectives

Our findings align with key theoretical perspectives on maltreatment and child development, offering insight into the mechanisms underlying the educational difficulties observed in our sample. The Developmental Psychopathology Framework (Cicchetti & Rogosch, 2002) underscores how early adversity influences multiple developmental domains over time, disrupting stress regulation and cognitive processing, which are crucial for learning. Children exposed to chronic maltreatment often develop attention difficulties and executive function impairments, making structured school environments particularly challenging (Clinchard et al., 2024; McLaughlin et al., 2014).

Emotional and behavioral difficulties may also contribute to school disengagement. Children with a history of maltreatment often develop heightened stress responses, social withdrawal, and fearfulness, which can manifest as school avoidance (Shonk & Cicchetti, 2001). Difficulties with self-regulation and cognitive flexibility may further interfere with learning, increasing the likelihood of requiring special education support (Bücker et al., 2012; Murgueitio et al., 2024). Additionally, frustration with academic tasks or negative school experiences may reinforce disengagement, compounding academic challenges over time (Skedgell & Kearney, 2018).

Attachment theory (Bowlby, 1988) provides a framework for understanding variability in school engagement among maltreated children. Some children may view school as a secure base, benefiting from consistent routines and supportive relationships with teachers, which help counteract the instability of home life (Chafouleas et al., 2019). However, others with insecure or disorganized attachment patterns may struggle to form trusting relationships with teachers and peers, leading to disengagement, avoidance, and absenteeism. This variability in attachment security could explain why some children in our study showed persistent absenteeism, while others remained engaged in school despite adversity. This is especially relevant for children exposed to multiple forms of maltreatment, who often lack safety and stability in their home environments. For them, school can serve as a uniquely stabilizing context, offering predictability and the opportunity to build positive connections. Within an attachment framework, such environments may partially compensate for the absence of secure relationships at home, highlighting the critical role of regular attendance. In this way, school becomes not only a place of learning but also a potential safe haven for the most vulnerable children.

The ecological systems perspective (Bronfenbrenner, 1979) highlights how school functioning is shaped not only by individual trauma histories but also by interactions within families, schools, and broader institutional structures. The significantly higher absenteeism rates in our sample compared to national norms suggest that beyond personal adversity, structural barriers—such as inadequate trauma-informed teacher training, inconsistent access to academic accommodations, and insufficient mental health services—may further contribute to school difficulties. For instance, children who experienced neglect may lack parental support for academic success, leading to long-term deficits in reading, writing, and overall school engagement (Manly et al., 2013). Additionally, frequent medical and support service appointments due to physical and mental health problems may further disrupt school attendance, exacerbating educational disparities.

Taken together, our findings suggest that an interplay of emotional, cognitive, academic, and systemic barriers contributes to school difficulties in maltreated children. Future research should aim to disentangle these mechanisms and develop targeted interventions that promote school attendance, engagement, and academic success for this vulnerable population. Strengthening trauma-informed educational practices, increasing school-based support systems, and equipping teachers with strategies to address the complex needs of maltreated children could help mitigate the long-term impact of early adversity on educational outcomes.

### Strengths and Limitations

A key strength of this study is its focus on a vulnerable group of children that is often difficult to recruit for research. By including only children with a documented history of maltreatment, the study allows for a detailed examination of the consequences of violence and abuse. This is particularly valuable as most research on maltreatment and educational outcomes is either retrospective or conducted with adolescents rather than primary school-aged children. Another strength is that multiple types of maltreatment were included, rather than examining only individual subtypes. Since maltreatment experiences often co-occur, this approach more accurately reflects real-world experiences and allows for a better understanding of the cumulative impact of exposure to multiple types of abuse.

The small sample size limited the types of analyses that could be conducted. For example, we were unable to explore potential sex differences or examine the impact of specific maltreatment subtypes separately. Some evidence suggests that certain types of maltreatment, such as neglect, may have a stronger impact on academic difficulties than others (Manly et al., 2013; McGuire & Jackson, 2018). Future studies with larger samples should examine these relationships in more detail.

Another limitation is the dichotomous classification of multivictimization as exposure to three or more types of maltreatment. This threshold was chosen primarily due to statistical power constraints, as a more detailed classification would have reduced our ability to detect meaningful associations. While this approach aligns with prior research on cumulative risk, definitions of multivictimization vary across studies, potentially affecting comparability. Future research should explore alternative thresholds and more refined classifications to improve consistency in defining and measuring multivictimization. A further limitation is the broad wording used to assess physical abuse (e.g., “physical violence or beaten up”), which may leave room for interpretation and underreporting. Some caregivers may not consider certain forms of corporal punishment, such as spanking, as abuse, leading to inconsistent reporting. Additionally, sibling abuse was not assessed, despite its relevance as a commonly overlooked form of maltreatment.

This study also relied on parent-reported school performance, which introduces potential subjectivity and bias. Since Norwegian primary schools do not assign grades, parental assessments were the most feasible option. However, caregivers’ perceptions of school performance may vary, particularly among those who have experienced adversity themselves. While some may overestimate their child’s abilities, others may underestimate difficulties, leading to potential bias in the reported data. A meta-analysis found that effect sizes were generally similar across subjective and objective assessments, but for emotional abuse, outcomes were more pronounced when reported subjectively (Qu et al., 2024). To address this limitation, we plan to link our dataset to national school registries in the future, once the children reach an age where grades are assigned, to complement the findings with objective school performance data.

Regarding generalizability, while the sample includes children from diverse socioeconomic backgrounds and family compositions, it may not be fully representative of all maltreated children. Participants were recruited from the Stine Sofie Center, meaning they had access to support services, which may set them apart from maltreated children who have not engaged with formal support systems. Future research should examine whether access to support services moderates the relationship between maltreatment and school outcomes, particularly in populations with varying levels of service availability.

Finally, foster children were not included in this study, which may limit the generalizability of our findings. The exclusion of foster children prevents direct comparisons of their school outcomes with those of maltreated children who remain with their biological families. It remains unclear whether foster children share similar sociodemographic characteristics, maltreatment histories, or academic challenges. Future research should explore whether their school functioning differs from that of non-fostered maltreated children.

### Practical and Clinical Implications

The findings highlight the need for targeted school support for children with a history of maltreatment. Educators and school staff must be equipped with the knowledge and tools to recognize and respond to the unique challenges these children face. Incorporating maltreatment awareness into teacher education programs and implementing trauma-informed practices in schools could help create more supportive learning environments (Dorado et al., 2016). Interventions to improve school functioning for maltreated children should focus on establishing safe and secure environments, providing behavioral support, and enhancing academic performance. Strategies such as teacher–child relationship building, structured classroom support, and parental involvement are effective (Fondren et al., 2020; Lowenthal, 2001). Engaging parents in interventions can be particularly beneficial, as supporting caregivers in promoting their child’s education may help mitigate the effects of early adversity. Collaboration between schools, health services, and social welfare agencies is crucial. Strengthening interdisciplinary and cross-sectoral cooperation has been identified as a key area for improvement in Norway’s ChildYouth21 strategy, which emphasizes the need for integrated support systems for children with a history of maltreatment.

Finally, enhancing academic achievement is critical not only for educational success but also for long-term mental health and employment outcomes (De Ridder et al., 2012). Ensuring that schools have the resources and strategies to support these children effectively is essential in breaking the cycle of adversity and promoting better life trajectories.

### Future Direction

Future research should prioritize longitudinal studies to track school outcomes over time and examine how maltreatment impacts academic trajectories across childhood and adolescence. This would provide a deeper understanding of how early adversity influences long-term educational and daily functioning. Using objective measures, such as standardized test scores and school records, would strengthen the validity of these findings and help disentangle the complex relationships between maltreatment, cognitive development, and academic performance. Further research should also explore early intervention strategies aimed at mitigating the negative effects of maltreatment on school functioning. Identifying protective factors that promote resilience and engagement in education could inform the development of targeted support programs to foster a more inclusive and supportive learning environment for these vulnerable children.

## Conclusion

This study highlights the educational vulnerabilities of children with a history of maltreatment, particularly in their early years of schooling. Many require special education, primarily due to academic difficulties, which are further compounded by multivictimization. Our findings show that exposure to multiple types of maltreatment is associated with increased school absences, raising the risk of long-term academic underachievement.

These results underscore the urgent need to better understand the effects of maltreatment and multivictimization on school functioning to develop more effective intervention and support strategies. Future research should continue to examine the long-term educational impacts of maltreatment and evaluate the effectiveness of intervention programs. Ensuring that maltreated children receive the necessary educational support is crucial in helping them succeed academically and overcome the challenges posed by early adversity.
